# Diets Rich in Saturated and Polyunsaturated Fatty Acids Induce Morphological Alterations in the Rat Ventral Prostate

**DOI:** 10.1371/journal.pone.0102876

**Published:** 2014-07-16

**Authors:** Angélica Furriel, Pamella Campos-Silva, Paola Cariello Guedes Picarote Silva, Waldemar Silva Costa, Francisco José Barcellos Sampaio, Bianca Martins Gregório

**Affiliations:** Department of Anatomy, State University of Rio de Janeiro, Urogenital Research Unit, Biomedical Center, Rio de Janeiro, Brazil; University of Cordoba, Spain

## Abstract

**Aim:**

To evaluate the influence of dietary lipid quality on the body mass, carbohydrate metabolism and morphology of the rat ventral prostate.

**Materials and Methods:**

Wistar rats were divided into four groups: SC (standard chow), HF-S (high-fat diet rich in saturated fatty acids), HF-P (high-fat diet rich in polyunsaturated fatty acids) and HF-SP (high-fat diet rich in saturated and polyunsaturated fatty acids). We analyzed body mass, fat mass deposits, plasma blood, insulin resistance and the ventral prostate structure.

**Results:**

Groups that received high-fat diets were heavier and presented larger fat deposits than SC group. The HF-S and HF-SP groups had higher glucose, insulin and total cholesterol serum levels and insulin resistance compared with the SC. The acinar area, epithelium height and area density of the lumen were higher in the HF-SP than in the other groups. The epithelium area density and epithelial cell proliferation were greater in the HF-P and HF-SP than in the SC group. All of the groups that received high-fat diets had greater area density of the stroma, area density of smooth muscle cells and stromal cell proliferation compared with the SC group.

**Conclusion:**

Diets rich in saturated and/or polyunsaturated fatty acids induced overweight. Independently of insulin resistance, polyunsaturated fatty acids increased prostate stromal and epithelial cell proliferation. Saturated fatty acids influenced only stromal cellular proliferation. These structural and morphometric alterations may be considered risk factors for the development of adverse remodeling process in the rat ventral prostate.

## Introduction

Obesity is the most common cause of insulin resistance (IR) in peripheral tissue as well as adipose tissue [1]. Obesity, IR and type 2 diabetes mellitus are considered risk factors for the development of benign prostatic hyperplasia (BPH) [2], [3]. BPH is the fourth most prevalent disease in the male population over the age of 50 years [4]; the etiology is multifactorial and may be affected by genetic [5], nutritional [6] and hormonal [7] factors. Experimental studies show that administering diets rich in lipids leads to the enlargement of the prostate in rats [3], [8]. Furthermore, obesity itself also contributes to the onset of BPH and many cancers, including prostate cancer [9], [10].

Previous studies have reported that polyunsaturated fatty acids-PUFAs (mainly eicosapentaenoic- EPA and docosahexaenoic-DHA), which are highly unsaturated, are more susceptible to lipid peroxidation. Lipid peroxides can increase the expression of the enzyme 5-alpha-reductase and consequently the formation of dihydrotestosterone (DHT), which could stimulate the growth of prostatic epithelial and stromal cells [11]. However, Liang and colleagues suggest that PUFAs such as alpha-linolenic acid and linoleic acid may act as potential endogenous inhibitors of the enzyme 5-alpha-reductase and thus as inhibitors of cell proliferation [12].

The action mechanism of saturated fatty acids (SFA) in prostate tissue is still controversial. Increased consumption of SFA increases the synthesis of total cholesterol and LDL-cholesterol and lowers HDL-cholesterol, increasing the risk for the development of BPH [2]. Van Kuilenburg and colleagues (2011) showed that dyslipidemia is associated with increased circulation of several growth factors, including basic fibroblast growth factor (bFGF) [13]. This growth factor acts as an important stimulator of fibroblast proliferation and collagen synthesis and deposition in the extracellular matrix and stimulates angiogenesis [14].

In light of these findings, it is important to evaluate the effects of obesity induced by administering different types of hyperlipidemic diets on the morphology of the rat ventral prostate.

## Materials and Methods

The animal protocols were approved by the Animal Ethics Committee of the State University of Rio de Janeiro (Protocol Number CEA027/2012), and the procedures were conducted in accordance with the guidelines for experimentation with animals (NIH Publication Nu. 85-23, revised 1996). The animals were housed at a controlled temperature (21±2°C) on a 12 h light/dark cycle with free access to food and water. Next, they were assigned to receive a specific diet.

### Experimental design

Thirty-nine 12-week-old male Wistar rats were divided into four groups. One group received only the standard chow throughout the entire experiment (SC group; n = 9), whereas the other groups received a high-fat diet (HF) classified according to its lipid content: HF-S (high-fat diet rich in saturated fatty acids; n = 10), HF-P (high-fat diet rich in polyunsaturated fatty acids; n = 10), HF-SP (high-fat diet rich in saturated and polyunsaturated fatty acids; n = 10). The SC diet (14% protein, 76% carbohydrate, 10% fat) and the high-fat diets (14% proteins, 36% carbohydrate, 50% fat) were prepared in accordance with the recommendations of the American Institute of Nutrition (AIN-93M) [15] (Table 1). Lard and/or canola oil was the source lipid of the diets. The diets were produced by *Pragsoluções* (Jau, SP, Brazil- www.pragsolucoes.com.br).

**Table 1:** Composition of experimental diets (following the AIN-93M recommendations for rodents) [18].

	SC	HF-S	HF-P	HF-SP
Corn starch	465.70	192.60	192.60	192.60
Casein	140.00	175.00	175.00	175.00
Sucrose	100.00	100.00	100.00	100.00
Soybean oil	40.00	40.00	40.00	40.00
**Rapeseed oil**	**0.00**	**0.00**	**238.00**	**119.00**
**Lard**	**0.00**	**238.00**	**0.00**	**119.00**
Fibre	50.00	50.00	50.00	50.00
L-cistin	1.80	1.80	1.80	1.80
Colin	2.50	2.50	2.50	2.50
Antioxidants	0.06	0.06	0.06	0.06
Mixed minerals	35.00	35.00	35.00	35.00
Mixed vitamins	10.00	10.00	10.00	10.00
TOTAL (g)	1000.00	1000.00	1000.00	1000.00
**Energy (KJ/Kg)**	**15925.80**	**20900.00**	**20900.00**	**20900.00**
Carbohydrate (%)	76.00	36.00	36.00	36.00
Protein (%)	14.00	14.00	14.00	14.00
**Lipid (%)**	**10.00**	**50.00**	**50.00**	**50.00**

*SC,* standard chow diet; *HF-S,* high-fat diet rich in saturated fatty acid (lard); *HF-P,* high-fat diet rich in polyunsaturated fatty acid (rapeseed oil); *HF-SP,* high-fat diet rich in saturated and polyunsaturated fatty acids.

The diets were administered to the rats from three to seven months of age. Each rat was weighed and measured weekly until the end of the experiment. All of the study groups received water and food *ad libitum,* and their food intake was assessed daily.

### Euthanasia

The animals were killed at 28-week-old. After 12 hours of fasting, the animals were deeply anesthetized (sodium pentobarbital intraperitoneally, 100 mg/kg), and all efforts were made to minimize suffering. Blood was collected directly from the left atrium. The ventral prostate was dissected and fixed for structural analyses. Epididymal, subcutaneous and retroperitoneal fat were also dissected, weighed and fixed.

### Serum biochemistry, hormone levels and carbohydrate metabolism

After the blood was collected, the serum was separated by centrifugation at room temperature (3000 rpm, 8 min) and stored at −20°C. Glucose, total cholesterol and triglyceride (TG) concentrations were measured using a colorimetric assay (Bioclin; Belo Horizonte, Minas Gerais, Brazil). An automatic spectrophotometer was used following the instructions recommended by the manufacturer of *Bioclin* commercial kits: glucose monoreagent-K082, cholesterol monoreagent-K083 and triglycerides monoreagent-K117. Serum analyses for insulin (Rat/Mouse Insulin kit, Millipore - Cat. EZRMI-13 k – St Charles, Missouri, USA) and testosterone (General Testosterone kit, Uscn - Cat. E90458Ge – Wuhan, China) were performed using commercially available enzyme-linked immunosorbent assay (ELISA) kits.

IR was calculated using the HOMA-IR (homeostasis model assessment for IR index): insulin*glucose/22.5.

### Immunohistochemistry

The immunohistochemical analyses were performed on the prostatic tissue from the ventral lobe of the rat prostate. Slides were prepared from 5-µm sections of the formalin-fixed, paraffin-embedded tissues, subjected to antigen retrieval with Tris-EDTA buffer (Proliferating Cell Nuclear Antigen- PCNA) and incubated with trypsin for 15 minutes at 37°C (Alpha Smooth Muscle Actin). Endogenous peroxidase activity was blocked by incubating the slides with 3% H_2_O_2_ in methanol for 15 minutes followed by applying a protein block (phosphate-buffered saline/bovine serum albumin- 5%). Mouse polyclonal primary antibodies to PCNA (1∶100; Invitrogen, 13-3900) and Alpha Smooth Muscle Actin (Invitrogen, 08-0106) were added and incubated overnight. Next, sections were treated with a biotinylated secondary antibody (K0679; Universal DakoCytomation LSAB Kit, Peroxidase, Glostrup, Denmark) and amplified with a biotin–streptavidin system (K0679; Universal DakoCytomation LSAB + Kit, Peroxidase, Glostrup, Denmark). 3, 3 diaminobenzidine tetrachloride (K3466, DakoCytomation, Glostrup, Denmark) was used as the chromogen. After incubation, the sections were counterstained with Mayer hematoxylin.

### Morphometric analysis

The prostate was dissected; fragments of the ventral lobe were fixed with freshly prepared fixative (1.27 M formaldehyde in 0.1 M phosphate buffer, pH 7.2) for 48 h at room temperature and embedded in Paraplast Plus (Sigma-Aldrich, St Louis, MO, USA). Next, the material was sectioned at a nominal thickness of 5 µm and stained with hematoxylin and eosin. For all of the morphometric analyses, five slides from each animal were obtained and five fields were evaluated for a total of 25 fields per animal. By performing an image analysis with a digital camera (Olympus DP71) on an Olympus BX51 microscope running ImageJ® software (Image Processing and Analysis in Java), the acinar area and the epithelium height were estimated using a minimum of 225 measures per group. For these analyses, we used photomicrographs at 200x and 600x magnifications, respectively.

The area density of the epithelium, lumen, connective tissue and smooth muscle cells (immunostained), expressed as a percentage, was estimated using the point intercepts method with a grid of 100 points superimposed on the magnified images (200x).

A quantitative assessment of proliferating cells was performed based on the anti-PCNA immunohistochemistry. Separately, the ratios of the number of dividing cell nuclei (immunostained) to the epithelial and stromal areas were calculated in each field we evaluated.

### Data analysis

The data were tested for normality and homogeneity of the variances and then reported as the means ± standard deviations (SDs). The differences among the groups were analyzed using a one-way analysis of variance (one-way ANOVA) followed by Bonferroni’s post hoc test. A *p-*value ≤0.05 was considered statistically significant (Prism version 5.00 for Windows; GraphPad Software, San Diego, California, USA).

## Results

### Food intake, Body mass evolution and fat deposits

There were no difference in the food intake among the SC (17.89±1.48 g), HF-S (16.42±2.59 g), HF-P (18.10±1.36 g) and HF-SP (16.45±5.10 g) groups. Throughout the experiment, the SC group was the lightest. At seven months of age, the body mass values of the HF-S (529.30±57.39 g), HF-P (546.40±40.13 g) and HF-SP groups (532.90±48.27 g) were 25%, 29% and 26% higher than the body mass of the SC group (424.22±40.29 g, *p*<0.0001), respectively (Table 2). It is important to mention that the animals subjected to the various HF diets had larger epididymal, retroperitoneal and subcutaneous fat deposits than the SC group. The epididymal fat mass of the HF-S, HF-P and HF-SP groups were 67%, 91% and 90% higher than that of the SC group (*p* = 0.0004), respectively. The retroperitoneal fat deposits of the HF-S, HF-P and HF-SP groups were 78%, 84% and 115% (*p* = 0.0001) greater, respectively, than the SC group, whereas the subcutaneous fat deposits were 181%, 121% and 204% (*p*<0.0001) greater compared with the SC group (Table 2).

**Table 2:** Biometric and metabolic parameters of the experimental groups.

Data	SC		HF-S		HF-P		HF-SP		ANOVA
	Mean	SD	Mean	SD	Mean	SD	Mean	SD	*p* value
Body mass, g	424.22	40.29	529.30	57.39**^a^**	546.40	40.13**^a^**	532.90	48.27**^a^**	***<0.0001***
Epididymal fat, mg	7.65	2.69	12.79	4.05**^a^**	14.64	4.75**^a^**	14.54	2.12**^a^**	***0.0004***
Retroperitoneal fat, mg	9.56	4.68	17.06	4.55**^a^**	17.56	2.73**^a^**	20.51	4.96**^a^**	***0.0001***
Subcutaneous fat, mg	2.48	1.37	6.96	1.61**^a^**	5.47	2.35**^a^**	7.55	1.42**^a^**	***<0.0001***
Total cholesterol, mg/dL	80.56	11.75	104.80	12.95**^a^**	100.10	10.38	105.20	19.65**^a^**	***0.002***
Triglycerides, mg/dL	86.29	23.68	95.63	8.60	83.63	17.52	84.25	5.04	0.37
Testosterone, ng/mL	5.48	0.83	4.73	1.14	4.28	1.25	5.11	0.89	0.25
Insulin, µlU/ml	1.49	0.41	2.75	0.45**^a^**	2.15	0.83	2.85	0.92**^a^**	***0.003***
Glucose, mmol/L	7.87	1.61	10.62	2.36**^a^**	9.85	1.61	11.09	1.65**^a^**	***0.006***
HOMA-IR	0.41	0.12	1.32	0.54**^a^**	0.91	0.37	1.36	0.50**^a^**	***0.003***

*SC,* standard chow diet; *HF-S,* high-fat diet rich in saturated fatty acid (lard); *HF-P,* high-fat diet rich in polyunsaturated fatty acid (rapeseed oil); *HF-SP,* high-fat diet rich in saturated and polyunsaturated fatty acids. The values are presented as the means and standard deviations (SD). The symbol [a] indicates a result that is different from the *SC* group (one-way ANOVA and Bonferroni’s post hoc test, *p*<0.05).

### Lipid profile, hormone levels and the HOMA-IR index

Triglyceride and testosterone levels were not different among the groups. However, the HF-S and HF-SP groups developed hypercholesterolemia at the end of the experiment. These two groups had plasma levels of total cholesterol that were 30% and 31% greater (p = 0.002), respectively, than the plasma levels of the SC group (Table 2). Besides, the HF-S and HF-SP groups developed hyperinsulinemia (*p* = 0.003) and hyperglycemia at the end of the experiment (*p* = 0.006) (Table 2). Consequently, these groups had higher HOMA-IR values than the SC group (*p* = 0.003) (Table 2). The HF-P group showed no differences in values of cholesterol, insulin, glucose and HOMA-IR index when compared to the SC, HF-S and HF-SP groups.

### Prostate morphometry

#### Acinar area and epithelial height

**Figure 1:**
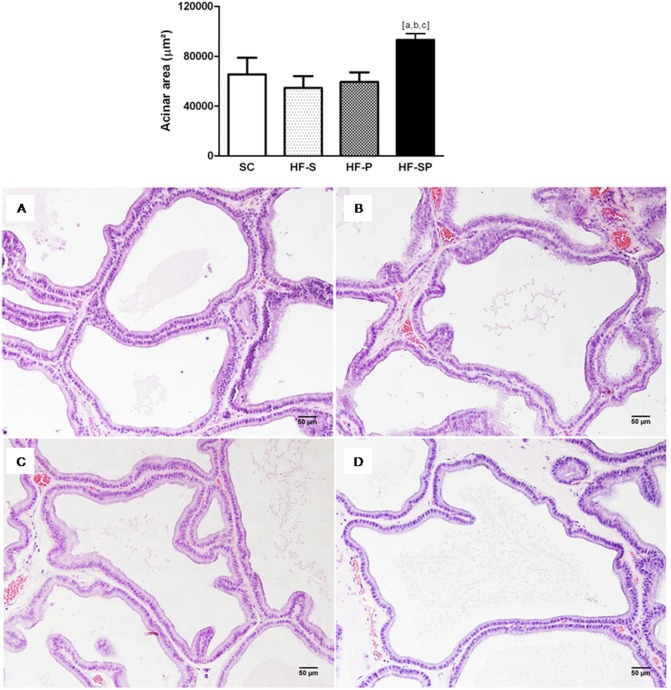
The acinar area changes caused by lard and canola oil on rat ventral prostate. *HF-SP* (a high-fat diet rich in saturated and polyunsaturated fatty acid) (D) resulted in a greater acinar area than the other diets. (A) *SC,* standard chow diet; (B) *HF-S,* a high-fat diet rich in saturated fatty acid (lard) and (C) *HF-P*, a high-fat diet rich in polyunsaturated fatty acid (canola oil). The symbol [a] indicates a result that is different from the *SC* group, [b] indicates a result that is different from the HF-S group and [c] indicates a result that is different from the HF-P group (one-way ANOVA and Bonferroni’s post hoc test, *p*<0.05). H&E staining, 200x.

**Figure 2:**
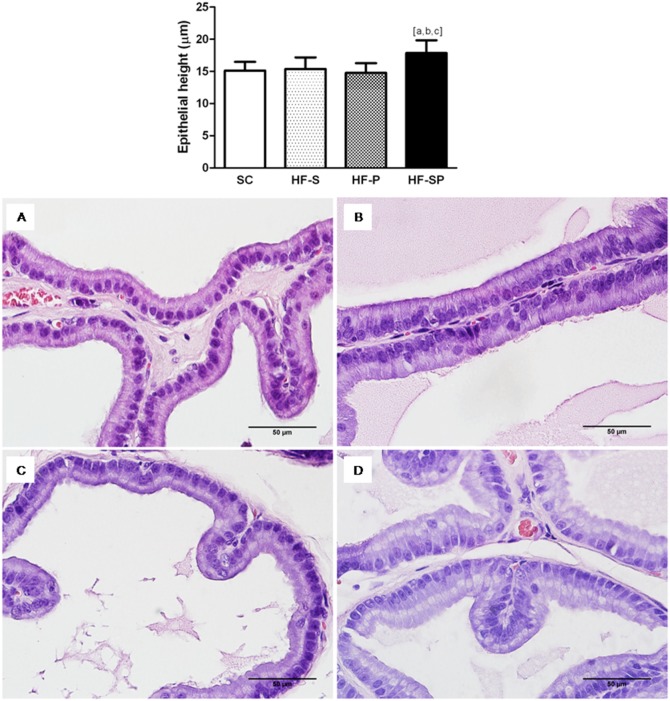
Epithelial height changes caused by lard and canola oil on rat ventral prostate. *HF-SP,* a high-fat diet rich in saturated and polyunsaturated fatty acid) (D) resulted in higher epithelial cell height than the other diets. (A) *SC,* standard chow diet; (B) *HF-S,* a high-fat diet rich in saturated fatty acid (lard) and (C) *HF-P,* a high-fat diet rich in polyunsaturated fatty acid (canola oil). The symbol [a] indicates a result that is different from the *SC* group, [b] indicates a result that is different from the HF-S group and [c] indicates a result that is different from the HF-P group (one-way ANOVA and Bonferroni’s post hoc test, *p*<0.05). H&E staining, 600x.

The HF-SP group showed an increase in the acinar area (SC: 65431±13278 µm^2^; HF-S: 54567±9439 µm^2^; HF-P: 59209±7801 µm^2^; HF-SP: 93078±16155 µm^2^; *p*<0.0001) (figure 1), as well as epithelial cell height (SC: 15.13±1.35 µm; HF-S: 15.38±1.79 µm; HF-P: 14.78±1.50 µm; HF-SP: 17.87±1.97 µm; *p* = 0.001) compared with the other groups (figure 2).

#### Area density: lumen, epithelium, connective tissue and smooth muscle cells

**Figure 3:**
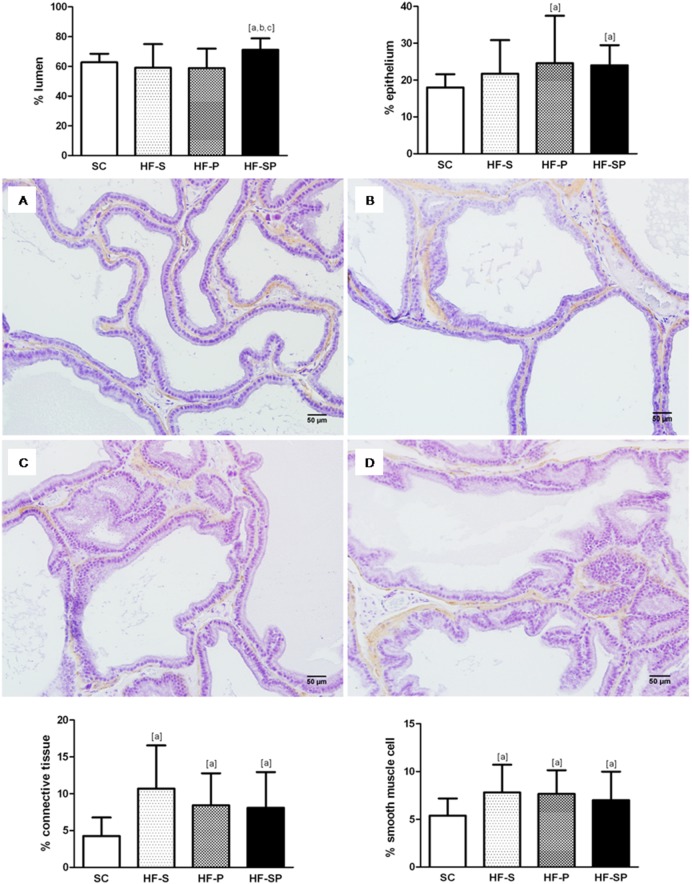
Morphological changes caused by different high-fat diets. (A) *SC,* standard chow diet produced no prostate alterations; (B) *HF-S,* a high-fat diet rich in saturated fatty acid (lard) caused an increase in the area density of the connective tissue and the smooth muscle cells; (C) *HF-P,* a high-fat diet rich in polyunsaturated fatty acid (canola oil) promoted an increase in the area density of the epithelium, the connective tissue and the smooth muscle cells; (D) *HF-SP,* a high-fat diet rich in saturated and polyunsaturated fatty acids induced an increase in the area density of the lumen, the epithelium, the connective tissue and the smooth muscle cells. The symbol [a] indicates a result that is different from the *SC* group, [b] indicates a result that is different from the HF-S group and [c] indicates a result that is different from the HF-P group (one-way ANOVA and Bonferroni’s post hoc test, *p*<0.05). H&E staining and immunostaining for Alpha Smooth Muscle Actin, 200x.

The lumen area density was greater in the HF-SP than the other groups (SC: 62.74±5.75%; HF-S: 59.00±15.97%; HF-P: 58.83±13.06%; HF-SP: 71.08±7.73%, *p*<0.0001). The HF-P and HF-SP groups showed an increased epithelium area density in comparison with the SC group (SC: 17.98±3.61%; HF-S: 21.72±9.13; HF-P: 24.59±12.84%; HF-SP: 24.01±5.48%, *p*<0.0001). In addition, all of the groups that received a high-fat diet had greater area density in the connective tissue (SC: 4.26±2.50%; HF-S: 10.69±5.87%; HF-P: 8.42±4.36%; HF-SP: 8.09±4.85%; p<0.0001) and in the smooth muscle cells (SC: 5.37±1.78%; HF-S: 7.79±2.91%; HF-P: 7.66±2.47%; HF-SP 7.01±2.98%, *p* = 0.0003) compared with the SC group (figure 3).

#### Epithelial and stromal cell proliferation

**Figure 4:**
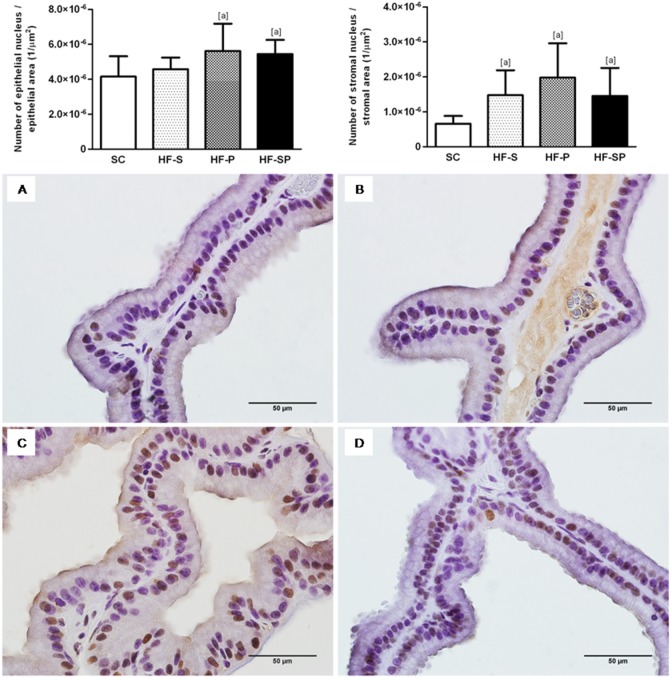
PCNA-positive cells in the epithelium and stroma of the rat ventral prostate. (A) *SC,* standard chow diet, (B) *HF-S*, high-fat diet rich in saturated fatty acid (lard), (C) *HF-P*, high-fat diet rich in polyunsaturated fatty acid (canola oil) and (D) *HF-SP,* high-fat diet rich in saturated and polyunsaturated fatty acids. The symbol [a] indicates a result that is different from the *SC* group (one-way ANOVA and Bonferroni’s post hoc test, *p*<0.05). Immunostaining for Proliferating Cell Nuclear Antigen- PCNA, 600x.

The HF-P and HF-SP groups had elevated epithelial cell proliferation in comparison with the SC group (SC: 4.15* 10^−6^±1.17* 10^−6^ 1/µm^2^; HF-S: 4.58* 10^−6^±6.59* 10^−7^ 1/µm^2^; HF-P: 5.62* 10^−6^±1.56* 10^−6^ 1/µm^2^; HF-SP: 5.44* 10^−6^±8.07* 10^−7^ 1/µm^2^; *p* = 0.0007). With regard to stromal cell proliferation, the groups that consumed a diet high in saturated and/or polyunsaturated fatty acids had higher values compared with the SC group (SC: 6.58* 10^−7^±2.23* 10^−7^ 1/µm^2^; HF-S: 1.47* 10^−6^±7.13* 10^−7^ 1/µm^2^; HF-P: 1.98* 10^−6^±9.79* 10^−7^ 1/µm^2^; HF-SP: 1.45* 10^−6^±8.05* 10^−7^ 1/µm^2^; *p* = 0.0003) (figure 4).

## Discussion

The present study evaluated the effects of obesity induced by different types of high-fat diets on the morphology of the rat ventral prostate. Our results show that diets rich in saturated fatty acids (HF-S), polyunsaturated fatty acids (HF-P) and both types of fatty acids (HF-SP) lead to overweight animals. The increase in body mass was confirmed by the increase in retroperitoneal, epididymal and subcutaneous fat deposits.

Recent studies have linked obesity with hyperinsulinemia and IR, which are considered risk factors for the development of metabolic syndrome [16]. Diets rich in SFA and cholesterol are associated with factors that negatively affect metabolism and predispose individuals to the development of IR and type 2 diabetes mellitus [17]. On the other hand, the consumption of PUFAs has been considered a protective factor against the development of these changes [17]. Our results align with these works because the animals in the HF-S and HF-SP groups showed IR, evidenced by the HOMA-IR values, as well as hyperinsulinemia and hyperglycemia. In this context, it is notable that consuming saturated fat (lard) in different concentrations (50% and 25% of the total energy of the diet) impaired carbohydrate metabolism, maximizing the damage to the prostate. However, a diet rich in PUFAs did not affect the glycemic response of the animals.

On a related note, as we expected, PUFAs inhibited the increase in serum cholesterol. Some studies recommend consuming PUFAs and MUFAs to improve the lipid profile [18], [19]. Inversely, excessive consumption of SFAs resulted in hypercholesterolemia, confirming the results previously reported in the literature [17]. Like Souza-Mello and colleagues (2007) [20] we did not find any differences in the plasma triglyceride levels of the groups, although foods rich in animal fat are associated with increased triglycerides [21].

However, the mechanism of SFAs and PUFAs act on prostate tissue is controversial and poorly understood. Our histomorphometric and immunohistochemical analyses showed that all of the animals that consumed a high-fat diet demonstrated a sharper proliferation in the stromal compartment of the prostate, along with increased area densities of the connective tissue and the smooth muscle cells. In addition, only the HF-P and HF-SP groups showed an increase in epithelial proliferation, which was confirmed by the increase in the area density of the epithelium. Numerous growth factors have been described as stimulators of stromal and epithelial cell proliferation; some of them act exclusively in the prostatic epithelium or stroma [22]. In our study, we found that PUFAs appear to stimulate the pathways that cause proliferation in both the epithelium and in the stroma, while the diets based on animal fats appear to be more related to proliferation in the stroma of the prostate.

Canola oil is rich in PUFAs of the n-3 series, which are precursors of long-chain PUFAs such as EPA and DHA that are more susceptible to lipid peroxidation. The lipid peroxides could increase the expression of the enzyme 5-alpha-reductase and consequently the formation of DHT [11]. Thus, they could generate cellular proliferation in both the epithelium and the stroma of the prostate, thereby triggering the expansion of the gland [10].

Hypercholesterolemic diets are associated with increased synthesis of bFGF, which can increase collagen production in the stromal compartment of the prostate. Van Kuilenburg and colleagues (2011) [13] observed that the increase in serum total cholesterol was positively correlated with an increase in circulating bFGF. Therefore, a diet high in cholesterol may increase the synthesis of bFGF and consequently induce proliferation solely in the stroma of the gland, as seen in our study.

Moreover, the enlargement of the prostate could be related with the increasing of testosterone serum levels [23]. However, like Vikram and colleagues (2010) [3], there were no significant changes in the circulating concentrations of testosterone in the different experimental groups which attribute the prostate growth to the diet administration.

Although the HF-P and HF-SP groups showed an increase in epithelial cell proliferation and an increase in the area density of the epithelium, only the HF-SP group showed an increase in the acinar area. This result was corroborated by the high area density of the lumen and the increase in the height of the epithelial cells. The increase in the epithelial area density observed in the HF-P group without a concomitant increase in the acinar area may indicate that there was an increase in the number of prostatic acini. Compared with the acini of the HF-SP group, the acini of the HF-P group may be smaller but more numerous. In contrast, the increase in the acinar area observed only in the HF-SP group can be explained by the increase in the size of the acinar epithelial cells. It has been suggested that hypertrophy of the prostatic epithelial cells is an indicator of the secretory capacity of these cells because the increase in secretion causes the dilation of organelles such as the endoplasmic reticulum and the Golgi complex [24]. Thus, diets rich in cholesterol do not appear to influence the proliferation of epithelial cells. However, they may be related to the stimulation of these secretory cells when combined with PUFAs, thus modifying the physiology of the gland.

It is noteworthy that the increase in cell proliferation in the group fed a high-fat diet based on canola oil was independent of the development of IR, showing the direct effect of PUFAs on the prostate. However, we cannot rule out the possibility that insulin stimulated cell proliferation, given that the HF-S and HF-SP groups were hyperglycemic and hyperinsulinemic (IR). Insulin may be associated with the pathogenesis of BPH through its excitatory action on the sympathetic nervous system [25]. It reduces the binding of globulin sex hormones, making these hormones bioavailable [26]. Their effects extend pathways mediated by insulin-like growth factor (IGF) [27]. Insulin can also stimulate prostate growth by activating pathways that trigger cellular proliferation, such as the IRS/PI3-K pathway which is associated with glucose uptake and the MEK/ERK pathway, which is responsible for mitogenic action. When IR develops, the IRS/PI3-K pathway is impaired while the MEK/ERK pathway remains unchanged [28].

The area density of smooth muscle increased in all of the animals receiving a high-fat diet, regardless of the lipid quality. The increase in this stromal component is closely associated with the pathogenesis of BPH. Studies in humans have shown that hyperplastic prostates had higher volume density of smooth muscle than normal prostates. The increase in the density and tone of these fibers may be related to the development of obstructive symptoms in BPH [29].

## Conclusions

Thus, the high-fat diet administration, independent of the lipid quality, promoted an increase of body mass and insulin resistance in animals. Polyunsaturated fatty acids increased stromal and epithelial cell proliferation. In contrast, saturated fatty acids influenced cellular proliferation in the stromal compartment only. Certainly, more studies are needed to confirm these findings, given the scarcity of the literature on the subject.
